# Anastomotic reinforcement in rectal surgery: a position statement from an expert panel of colorectal surgeons

**DOI:** 10.1007/s13304-026-02755-y

**Published:** 2026-08-31

**Authors:** Arcangelo Picciariello, Alfredo Annicchiarico, Rossella Melcarne, Donato Francesco Altomare, Alberto Arezzo, Paolo Pietro Bianchi, Marco Caricato, Massimo Carlini, Claudio Coco, Francesco Corcione, Paolo Delrio, Gaetano Luglio, Marco Milone, Massimiliano Mistrangelo, Corrado Pedrazzani, Daniela Rega, Antonino Spinelli, Gaya Spolverato, Leonardo Vincenti, Massimo Giuseppe Viola, Gianpiero Gravante

**Affiliations:** 1 Department of Experimental Medicine, University of Salento, Lecce, Italy; 2 Department of Surgery, Azienda Sanitaria Locale Di Parma, Vaio Hospital, Fidenza, Italy; 3 Department of Translational and Precision Medicine, Sapienza University of Rome, Rome, Italy; 4 Dipartimento di Medicina di Precisione e Rigenerativa e Polo Jonico (DiMePRE-J), Università degli Studi Aldo Moro di Bari, Bari, Italy; 5 Department of Surgical Sciences, University of Torino, Turin, Italy; 6 Department of Health Sciences, University of Milan, Milan, Italy; 7 Department of General and Robotic Surgery, ASST Santi Paolo e Carlo, Milan, Italy; 8 UOC Chirurgia Colorettale, Fondazione Policlinico Universitario Campus Bio-Medico di Roma, Via Àlvaro del Portillo 200, 00128 Rome, Italy; 9 General Surgery Unit, Department of General Surgery, S. Eugenio Hospital, Rome, Italy; 10 General Surgery 2 Unit, Fondazione Policlinico Universitario Agostino Gemelli IRCCS, 00168 Rome, Italy; 11 Clinica Mediterranea, Via Orazio, 2, 80122 Naples, Italy; 12 Colorectal Surgical Oncology, Abdominal Oncology Department, Istituto Nazionale per lo Studio e la Cura dei Tumori, “Fondazione G. Pascale” IRCSS, Naples, Italy; 13 Coloproctology Unit, Department of Public Health, University of Naples “Federico II”, Naples, Italy; 14 Department of Clinical Medicine and Surgery, “Federico II” University of Naples, Via Pansini 5, 80131 Naples, Italy; 15 Surgical Science Department, Centre of Minimal Invasive Surgery, Città Della Salute E Della Scienza Hospital, University of Turin, Turin, Italy; 16 Unit of General Surgery 1, CISMed - Centre for Medical Sciences, Trento University, Santa Chiara Hospital – APSS, Trento, Italy; 17 Department of Biomedical Sciences, Humanitas University, Via Rita Levi Montalcini 4, 20072 Pieve Emanuele, Milan, Italy; 18 General Surgery 3, Department of Surgical, Oncological and Gastroenterological Sciences (DiSCOG), University of Padova, Padua, Italy; 19 Deparment of Surgery, I.R.C.C.S. Saverio de Bellis, Castellana Grotte, Italy; 20 Department of General, Robotic, Oncologic Surgery, G. Panico Hospital, Tricase, LE Italy; 21 Department of General Surgery, Azienda Sanitaria Locale ASL Lecce, Casarano, Italy

## Abstract

Anastomotic leakage (AL) remains one of the most feared complications after rectal surgery for cancer, with stable incidence rates despite advances in surgical technique and perioperative care. Multiple anastomotic reinforcement strategies have been proposed, yet evidence remains heterogeneous and no clear consensus exists regarding their routine use. A structured expert position panel process was conducted. Eleven clinical scenarios based on AL risk factors and twenty thematic items addressing indications, devices, timing, contraindications, surgical approach, stoma use, costs, and expected outcomes were developed. A panel of experts evaluated each statement using a five-point Likert scale through a two-round process. Consensus was assessed using median scores and interquartile ranges (IQR). Consensus was not reached for most individual or combined preoperative risk factors. Most reinforcement techniques did not receive clear endorsement because of inconsistent and heterogeneous evidence. Strong agreement emerged regarding the central role of intraoperative assessment: tissue perfusion, mechanical tension, tissue quality, and unexpected technical difficulty were identified as key determinants. The panel agreed that reinforcement may still be appropriate in the presence of a diverting stoma, as diversion reduces clinical severity but does not prevent leakage or long-term sequelae. Surgical approach and material cost were not considered influent factors. This position statement highlights the lack of robust evidence supporting routine, indication-driven, or device-specific anastomotic reinforcement in rectal cancer surgery. The main shared recommendation was to base reinforcement on intraoperative surgical judgment, including tissue perfusion, tension, tissue quality, and unexpected technical difficulty.

Keywords: anastomotic leakage, rectal cancer surgery, anastomotic reinforcement

## Introduction

Anastomotic leakage (AL) represents a serious and particularly feared complication following rectal resection for cancer, accounting for considerable rates of morbidity (20–35%) and mortality (2–16%) [1–3]. Although significant progress has been achieved in surgical techniques, perioperative care, and patient selection, the overall incidence of AL has remained relatively stable, with rates ranging from 3–8% [4], and even higher percentages reported in cases of low rectal resections [5]. Male sex consistently been identified in large cohort studies and multivariate analyses as an independent risk factor for AL [6–8], with reported odds between 1.56 and 3.62 [8–10], due to the narrower male pelvis and consequent technical difficulty [8, 11]. However, the risk of leakage is cumulative and interaction-dependent rather than driven by isolated elements. Large multicenter Korean analyses, demonstrate that male sex, low rectal tumor, neoadjuvant chemoradiotherapy, transfusion, technically difficult pelvic dissection, and multiple stapler firings independently and jointly increase leak risk after rectal resection [8].

The persistently high incidence has prompted the development and clinical evaluation of multiple strategies aimed at reinforcing the anastomosis and reducing the risk of leakage. Over the past two decades, different approaches have been proposed, including mechanical, biological, and other reinforcement techniques [12, 13]. Some studies have addressed single methods (e.g., fibrin sealants, reinforced sutures), whereas others grouped a wide range of interventions together, limiting the comparability and synthesis of evidence [14–17]. Overall, the current literature is characterized by heterogeneity in study design, patient populations, outcome definitions, and clinical endpoints. Existing systematic reviews summarize heterogeneous evidence on individual reinforcement strategies [12, 13], but they do not clarify how surgeons should integrate this evidence into intraoperative decision-making.

This position statement was therefore designed to identify practical areas of agreement, uncertainty, and disagreement among experienced rectal surgeons, with particular focus on real-time intraoperative assessment rather than device-driven recommendations.

## Materials and methods

A working group (AP, RM, AA, and GG) conducted a comprehensive literature review to gather all available evidence regarding anastomotic reinforcement strategies. Searches were performed across four major electronic databases: PubMed/MEDLINE, Embase, Scopus, and the Cochrane Database of Systematic Reviews. Both controlled vocabulary and free-text terms related to colorectal anastomosis and reinforcement techniques were used in combination. The search strategy included: ("colorectal surgery" OR "colorectal anastomosis") AND ("reinforcement" OR "buttress" OR "sealant" OR "mesh" OR "fibrin glue" OR "patch" OR "wrap") AND ("systematic review" OR "meta-analysis"). Eligible studies included randomized controlled trials, prospective and retrospective cohort studies, case series, systematic reviews, and meta-analyses. The review process focused on the following reinforcement techniques: transanal tube (TAT), reinforced handsewn sutures, biological reinforcements (TachoSil®, Hemopatch®), intraluminal barrier devices (C-Seal™), extraluminal protection devices (Colovac®), and reinforced staplers (Seamguard®).

After the review process, the working group developed 11 clinical scenarios based on the presence of one or more risk factors, together with an additional 20 items covering five thematic areas: clinical indications; techniques and devices; timing; contraindications; and stoma, surgical approach, strategy, and expected outcomes. The clinical scenarios were created based on the largest multicenter analysis of risk factors for anastomotic leakage after rectal cancer excision [8]. Each scenario was pragmatically selected to reflect the most common and clinically relevant combinations of risk factors encountered in daily practice, and was subsequently translated into an individual statement/item.

Panel members were purposively selected by the working group to include surgeons with recognized experience in rectal cancer surgery, documented scientific activity, and involvement in high-volume colorectal or oncologic surgical practice. The aim was not to obtain a statistically representative national sample, but to assemble a clinically credible expert panel able to discuss real-world decision-making in an area where evidence remains heterogeneous. The purpose of the study was explained and, after agreement, each individual scenarios/item was submitted to panel members through an online survey administered via Google Forms® (Google LLC, Mountain View, CA, USA) using an email link. Expert members of the panel contributed by providing structured feedback on the survey items, and participating in the iterative refinement process. The estimated completion time was approximately 20 min, and only one response per participant was allowed. All questions were mandatory to prevent missing data.

The process followed a structured, two-round iterative design with controlled feedback between rounds. The working group designed the study, reviewed the literature, developed the survey, analyzed responses, and drafted the manuscript. Panel members completed both survey rounds, reviewed aggregated results, provided structured comments on the interpretation of findings, critically revised the manuscript, and approved the final version. This study was designed as a structured expert position panel, incorporating anonymous rating, controlled feedback, and descriptive aggregation of responses. It was not intended to represent a formal Delphi, RAND/UCLA, or guideline development process.

### Statistical analysis

The web-based platform automatically collected responses and exported them into a Microsoft Excel spreadsheet. For each statement, the median Likert score was used to describe the overall direction of the panel’s judgment, indicating the degree of agreement or disagreement with the statement. Participants rated each scenario or item using a five-point Likert scale: 5 = strongly agree; 4 = agree; 3 = neutral; 2 = disagree; 1 = strongly disagree. For descriptive purposes, the distribution of responses across each Likert category was reported as percentages.

The interpretation of each statement was based on the combined assessment of the median value, which expressed the panel’s overall position, and the interquartile range (IQR), which quantified the degree of agreement among experts. Given the ordinal nature of Likert-scale data, responses were summarized using medians and interquartile ranges. Lower median values (1–2) indicated disagreement with the statement, whereas higher median values (4–5) indicated agreement; a median value of 3 was interpreted as neutrality, reflecting no clear preference toward the statement. The interquartile range (IQR) was used to describe the degree of convergence among panel members, without implying formal Delphi consensus thresholds. The IQR was used independently to assess consensus strength among experts, with smaller values (0.5–1.0) indicating greater homogeneity of responses and stronger consensus, larger values (2.0–2.5) reflecting increased variability and weaker consensus, and an IQR of 1.5 indicating borderline consensus. Only statements showing borderline consensus (IQR = 1.5) were resubmitted for the second round. Percentages for each response category were reported descriptively.

## Results

The entire process was conducted between August and December 2025. The list of scenarios and items presented to the experts, the results expressed as percentages according to the Likert scale, and the levels of agreement achieved in the first and second rounds are reported in Tables 1 and 2. All experts completed the questionnaires in both rounds.

**Table 1:** Statements submitted to experts and responses expressed as percentages of agreement according to the five-point Likert scale

Section	Item / scenario		Strongly agree	Agree	Neutral	Disagree	Strongly disagree
Clinical indications	1	Perioperative blood transfusion (Hazard Risk 10.705): A 74-year-old woman undergoes low anterior resection for a rectal adenocarcinoma located 9 cm from the anal verge. She did not receive neoadjuvant therapy. During surgery, bleeding from the inferior mesenteric artery required intraoperative hemostasis and transfusion of two units of packed red blood cells. Knight-Griffen anastomosis is performed.*	11.8%	29.4%	23.5%	29.4%	5.9%
	2	Neoadjuvant chemoradiotherapy (Hazard Risk 6.284): A 61-year-old man with a rectal tumor at 9 cm from the anal verge completes long-course neoadjuvant chemoradiotherapy. He subsequently undergoes low anterior resection with Knight-Griffen anastomosis. No transfusions or other perioperative complications are recorded.*	11.8%	29.4%	11.8%	23.5%	23.5%
	3	≥ 3 linear stapler firings (Hazard Risk 6.181): A 72-year-old woman undergoes rectal resection for a tumor located 10 cm from the anal verge. No neoadjuvant therapy was administered. Due to anatomical constraints, three or more linear stapler cartridges are required to transect the rectum. The Knight-Griffen technique is used.*	5.9%	52.9%	11.8%	23.5%	5.9%
	4	Male sex (Hazard Risk 3.468): A 59-year-old man undergoes low anterior resection for a rectal tumor at 8 cm from the anal verge. No neoadjuvant therapy or transfusions. ASA II. The Knight-Griffen technique is used.*	0%	17.6%	47.1%	23.5%	11.8%
	5	Tumor < 7 cm from anal verge (Hazard Risk 2.418): A 70-year-old woman undergoes resection for a rectal cancer located 6.5 cm from the anal verge. No neoadjuvant therapy or intraoperative issues are noted. Knight-Griffen anastomosis is performed.*	5.9%	41.2%	23.5%	17.6%	11.8%
	6	Male + neoadjuvant chemoradiotherapy (Hazard Risk 3.851): A 58-year-old man undergoes low anterior resection for rectal cancer at 9 cm from the anal verge after completing neoadjuvant chemoradiotherapy. The Knight-Griffen technique is used. No perioperative transfusions.*	17.6%	41.2%	11.8%	17.6%	11.8%
	7	Tumor < 7 cm + neoadjuvant chemoradiotherapy (Hazard Risk 3.471): A 64-year-old woman with a tumor at 6 cm from the anal verge undergoes low anterior resection after long-course neoadjuvant chemoradiotherapy. Knight-Griffen anastomosis is performed. No transfusions or additional risk factors are present.*	11.8%	41.2%	11.8%	23.5%	11.8%
	8	Neoadjuvant chemoradiotherapy + ≥ 3 stapler firings (Hazard Risk 3.184): A 69-year-old man with a rectal tumor at 8 cm from the anal verge completes neoadjuvant chemoradiotherapy. During surgery, four stapler cartridges are required to divide the rectum. Anastomosis is performed using Knight-Griffen technique.*	23.5%	35.3%	0%	35.3%	5.9%
	9	Tumor < 7 cm + ≥ 3 stapler firings (Hazard Risk 2.104): A 75-year-old woman with a tumor at 6.2 cm from the anal verge undergoes resection without neoadjuvant therapy. Four linear stapler firings are required due to technical difficulty. Knight-Griffen anastomosis is completed.*	35.3%	29.4%	11.8%	17.6%	5.9%
	10	Male + ≥ 3 stapler firings (Hazard Risk 1.758): A 67-year-old man undergoes rectal resection for a tumor at 8 cm from the anal verge. No neoadjuvant therapy. Four stapler cartridges are needed for rectal division. Knight-Griffen anastomosis is performed.*	0%	41.2%	11.8%	17.6%	29.4%
	11	Neoadjuvant chemoradiotherapy + Tumor < 7 cm + ≥ 3 stapler firings: A 70-year-old woman with a rectal adenocarcinoma at 6 cm from the anal verge completes long-course neoadjuvant chemoradiotherapy. The patient refuses the creation of a diverting stoma after thorough explanation of potential risks. During low anterior resection, difficult pelvic access requires the use of four linear stapler cartridges to transect the rectum. Knight-Griffen anastomosis is performed.*	47.1%	23.5%	5.9%	17.6%	5.9%
Techniques/devices	12	Transanal tube (TAT)	11.8%	29.4%	23.5%	29.4%	5.9%
	13	Reinforced hand-sewn sutures	29.4%	41.2%	23.5%	0.0%	5.9%
	14	Biological reinforcement (e.g., TachoSil®, Hemopatch®)	5.9%	11.8%	29.4%	41.2%	11.8%
	15	Intraluminal barrier device (C-Seal™)	0%	5.9%	70.6%	23.5%	0.0%
	16	Extra-luminal protection device (Colovac®)	0%	5.9%	64.7%	29.4%	0.0%
	17	Reinforced stapler (Seamguard®)	0.0%	29.4% (0%)	47.1% (58.8%)	17.6% (35.3%)	5.9%
Timing	18	The decision to reinforce an anastomosis can be made intraoperatively based on surgical difficulty or poor tissue quality, even in the absence of known risk factors	29.4%	52.9%	5.9%	11.8%	0.0%
	19	Intraoperative signs of suboptimal perfusion or difficult access should prompt the use of reinforcement techniques	17.6%	41.2%	11.8%	23.5%	5.9%
	20	Anastomotic reinforcement should be performed after completion of the anastomosis and after the leak test	35.3% (17.6%)	41.2% (52.9%)	11.8%	11.8%	0% (5.9%)
Controindications	21	There are no absolute contraindications to the use of anastomotic reinforcement in rectal surgery	11.8%	70.6%	17.6%	0.0%	0.0%
	22	Infection, peritonitis, or tissue necrosis are absolute contraindications to reinforcement	17.6%	17.6%	35.3%	23.5%	5.9%
	23	Pelvic edema or tissue fragility represent relative contraindications to reinforcement	5.9% (0%)	17.6%	35.3% (23.5%)	35.3% (52.9%)	5.9%
Stoma	24	The use of an anastomotic reinforcement technique is appropriate even when a diverting stoma is planned	11.8%	47.1% (70.6%)	17.6% (11.8%)	23.5% (5.9%)	0.0%
	25	When a diverting stoma is created, reinforcement provides no additional clinical benefit	0%	5.9%	35.3%	52.9%	5.9%
Surgical approach	26	The indication for anastomotic reinforcement should be consistent regardless of the surgical approach (open, laparoscopic, robotic)	41.2%	47.1%	0%	11.8%	0.0%
	27	Robotic surgery reduces the need for anastomotic reinforcement due to improved precision	5.9%	11.8% (5.9%)	17.6% (11.8%)	41.2% (52.9%)	23.5%
Selective vs. systematic strategy	28	Anastomotic reinforcement should be reserved for patients with clearly defined risk factors for anastomotic leakage	5.9%	35.3%	41.2%	17.6%	0.0%
	29	The cost of reinforcement materials limits their use to selected high-risk cases	0%	11.8%	17.6%	58.8%	11.8%
Expected outcomes	30	Reinforcement reduces the rate of clinically relevant anastomotic leakage and the need for reintervention or radiological drainage procedures	5.9%	41.2%	35.3%	17.6%	0.0%
	31	Reinforcement shortens hospital stay and improves short-term outcomes	5.9%	23.5%	29.4%	35.3%	5.9%

Percentages represent results obtained during the 1st round, those in brackets changes recorded after the 2nd round. * In the following scenario, I would apply an anastomotic reinforcement or preventive method, assuming that no ileostomy is performed. Hazard risk refers to Park et al. [8]

**Table 2:**
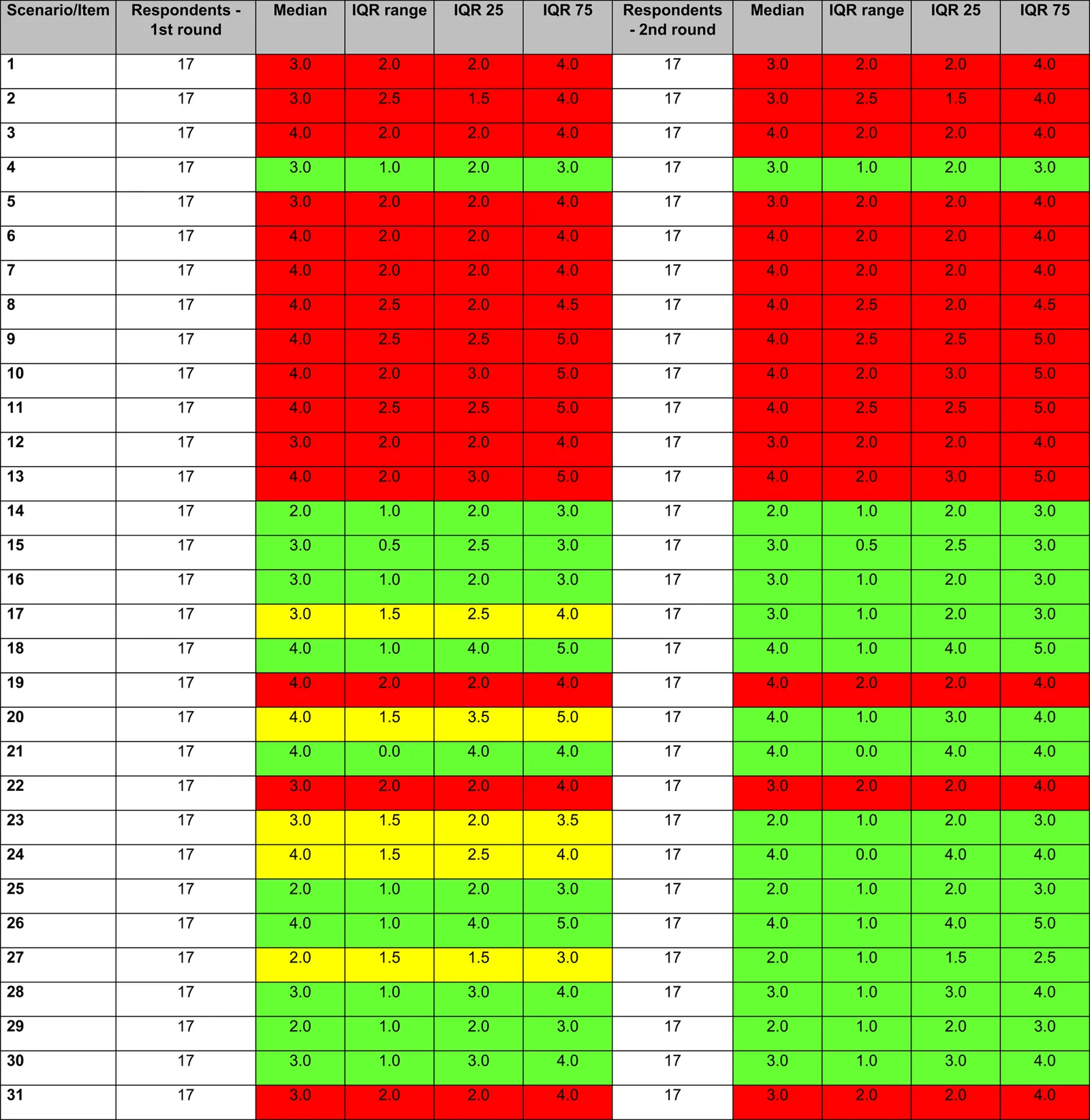
Results analyzed and expressed as median and interquartile range (IQR) across the first and second rounds

Red indicates scenarios with no agreement, yellow indicates borderline agreement (these questions proceeded to a second round), and green indicates scenarios in which agreement was achieved (either during the first or second round). IQR 25: 25° interquartile, IQR 75: 75° interquartile

### Clinical indications (scenarios 1–11)

Table 1 summarizes the distribution of responses for the 11 clinical scenarios. Overall, no consistent agreement emerged supporting reinforcement based exclusively on individual preoperative risk factors or predefined combinations of risk factors. When considering single risk factors, the panel did not reach consensus toward reinforcement for perioperative transfusions (Scenario 1), neoadjuvant chemoradiotherapy (Scenario 2), greater than 3 stapler firings (Scenario 3), tumor less than 7 cm from the anal verge (Scenario 5; Tab.1). The only single risk factor where the panel reached a consensus about reinforcement methods, albeit towards neutrality, was male sex (Scenario 4).

When two risk factors were combined, again no consensus was reached: male + neoadjuvant therapy (Scenario 6), tumor < 7 cm + neoadjuvant therapy (Scenario 7), combinations involving ≥ 3 stapler firings when paired with neoadjuvant therapy (Scenario 8), tumor height < 7 cm (Scenario 9), male sex (Scenario 10). Finally, three major risk factors were not sufficient to reach a consensus i.e. neoadjuvant chemoradiotherapy, tumor < 7 cm, and ≥ 3 stapler firings (Scenario 11).

### Techniques/devices (items 12–17)

No reinforcement technique achieved strong endorsement from the panel. TAT and reinforcement sutures generated favorable tendencies but failed to achieve sufficient convergence. Biological reinforcements were the only category reaching agreement toward disagreement, whereas C-Seal™, Colovac®, and reinforced staplers resulted in neutral positions.

### Intraoperative decision-making and timing of reinforcement (items 18–20)

Intraoperative decision-making generated the strongest agreement observed throughout the survey. Experts consistently supported reinforcement when guided by intraoperative assessment and agreed that reinforcement should be considered after completion of the anastomosis and leak testing. However, no agreement was reached regarding specific intraoperative findings as absolute indications for reinforcement.

### Contraindications (items 21–23)

Panel members generally did not identify clear contraindications to anastomotic reinforcement. Agreement was reached that reinforcement has no absolute contraindications (Item 21), whereas infection, peritonitis, tissue necrosis, pelvic edema, and tissue fragility failed to generate consistent support as absolute or relative reasons to avoid reinforcement.

### Stoma (items 24–25)

Strong agreement emerged that reinforcement may remain appropriate even when a diverting stoma is planned. Conversely, experts disagreed with the statement that diversion alone eliminates any potential role for reinforcement.

### Surgical approach (items 26–27)

Experts agreed that indications for reinforcement should not depend on whether surgery is performed through an open, laparoscopic, or robotic approach. Consistently, robotic surgery was not considered sufficient justification to reduce or omit reinforcement strategies.

### Selective vs. systematic strategy (items 28–29)

Responses did not support a purely risk factor-driven reinforcement strategy. Experts adopted a neutral position regarding the restriction of reinforcement to predefined high-risk patients and disagreed that material costs should represent a major determinant of reinforcement use.

### Expected outcomes (items 30–31)

Experts did not reach a shared position regarding the ability of reinforcement to reduce clinically relevant leakage, reinterventions, hospital stay, or short-term outcomes. Although reinforcement was generally viewed as a potentially useful adjunct, its clinical impact was considered insufficiently established.

## Discussion

The main finding of this expert position panel is not the endorsement of a specific reinforcement strategy, but the recognition that anastomotic reinforcement in rectal surgery is primarily guided by intraoperative surgical judgment. In a field characterized by heterogeneous and limited comparative evidence, experts did not reach agreement on reinforcement based either on predefined risk profiles or on specific reinforcement technologies. Notably, the lack of agreement persisted not only for individual risk factors but also for progressively combined risk scenarios, suggesting that experienced rectal surgeons do not routinely translate established epidemiological predictors of AL into reinforcement decisions, even in high-risk scenarios with multiple risk factors.

Similarly, the absence of consensus regarding specific reinforcement methods likely reflects the heterogeneous and frequently conflicting evidence available in the literature. Although some techniques, including TAT and reinforcement sutures, have shown promising results in selected studies [12, 18–22], randomized trials and comparative analyses have generally failed to demonstrate consistent and reproducible reductions in clinically relevant leakage [23]. Biological sealants, intraluminal devices, extraluminal protection systems, and reinforced staplers remain supported mainly by limited or conflicting evidence [24–35]. Consequently, panel members did not endorse any single reinforcement strategy as a standard component of rectal surgery.

Instead, operative findings appear to play a more influential role in clinical decision-making. Several determinants of anastomotic healing, including tissue perfusion, mechanical tension, tissue quality, and technical difficulty [36, 37], become fully apparent only during surgery [38–40]. Consistently, strong agreement emerged regarding the importance of intraoperative assessment, with tissue quality, perfusion, mechanical tension, and unexpected technical difficulties being regarded as the most relevant factors when considering reinforcement. Interestingly, agreement was achieved for the principle of intraoperative judgment (Item 18), but not for specific intraoperative triggers such as impaired perfusion or difficult access (Item 19). Again, this suggests that surgeons support individualized decision-making rather than predefined operative algorithms.

Additional areas of agreement further support the pragmatic nature of current reinforcement practice. The panel considered reinforcement potentially useful even in the presence of a diverting stoma, as AL is associated in the long term with stenosis, chronic fistulas, intestinal dysfunction, incontinence, and impaired quality of life regardless of the presence of a diverting ileostomy [41], and recognized that diversion reduces the clinical consequences of leakage without preventing leakage itself [42]. Surgical approach was not considered a determinant of reinforcement strategy, as available evidence does not demonstrate meaningful differences in leak rates between open, laparoscopic, and robotic platforms [43, 44]. Finally, economic considerations were not viewed as a major limiting factor, since the clinical and financial burden associated with AL substantially exceeds the cost of currently available reinforcement materials [45]. AL is associated with approximately seven additional hospital days, ICU admissions, and over $24,000 per leak episode [46, 47]. Formal cost-effectiveness data on reinforcement techniques remain limited and are largely model-based [35], but the available evidence suggests a net benefit compared with the costs of managing AL. For example, the use of fibrin sealant to prevent AL reduced the average cost per case by approximately €900, despite the additional material expense [48].

Overall, the panel findings suggest that current reinforcement strategies should be regarded as selective adjuncts rather than standardized interventions. The absence of consensus for specific devices or predefined risk profiles does not imply lack of utility, but rather reflects the limited certainty of the available evidence and the continued importance of individualized intraoperative decision-making. This study however has some limitations, and should not be interpreted as a formal Delphi consensus or guideline. The panel was purposively selected, relatively small, and composed of surgeons practicing in Italy; therefore, the findings may not be generalizable. In addition, the process used descriptive thresholds based on median and IQR rather than predefined Delphi consensus criteria. These limitations are inherent to expert position statements and reflect the exploratory nature of the work.

## Conclusions

A consistent agreement emerged on the central role of intraoperative surgical judgment. Real-time assessment of tissue perfusion, mechanical tension, tissue quality, and unexpected technical difficulty represents the most reliable framework to guide decisions on anastomotic reinforcement. Conversely, no shared position supported routine, indication-driven, or device-specific use of reinforcement in rectal cancer surgery. Overall, these findings support a pragmatic, condition-based approach in which reinforcement is not dictated by predefined risk thresholds or specific technologies, but rather tailored to intraoperative findings and surgeon expertise. Accordingly, current reinforcement strategies should be considered adjunctive and selective, rather than standardized components of rectal surgery.

## Data Availability

The data generated and analyzed from the expert panel voting process were collected and analyzed for the purposes of this position statement. The underlying data are available from the corresponding author upon reasonable request.
